# A prognosis marker MUC1 correlates with metabolism and drug resistance in bladder cancer: a bioinformatics research

**DOI:** 10.1186/s12894-022-01067-8

**Published:** 2022-07-25

**Authors:** Liangliang Qing, Qingchao Li, Yongjin Yang, Wenbo Xu, Zhilong Dong

**Affiliations:** 1 Gansu Nephro-Urological Clinical Center, Institute of Urology, Department of Urology, Key Laboratory of Urological Disease of Gansu Province, Lanzhou University Second Hospital, Lanzhou, China; 2 Department of Urology, Lanzhou University Second Hospital, No. 82, Cuiyingmen, Chengguan District, Lanzhou, 730030 Gansu China

**Keywords:** Bladder cancer, MUC1, Metabolism, Immune, Drug resistance, Prognosis

## Abstract

**Background:**

MUC1 is a type I transmembrane protein that plays an important role in tumor cell signal transduction. Although current studies have shown that MUC1 is upregulated in bladder cancer (BC), the specific mechanism is still unclear.

**Methods:**

We performed expression analysis, gene set enrichment analysis, survival analysis, immune infiltration analysis, drug sensitivity analysis, and metabolism-related gene expression analysis on TCGA-BLCA, GES31684 and GSE13507.

**Results:**

The expression of MUC1 in the tumor and lymphatic metastasis positive samples was significantly increased. Genes related to MUC1 expression were significantly enriched in immune response, ribosomes, exosomes, and energy metabolism. The results of the immune infiltration analysis showed that M1 macrophages in BC with high MUC1 expression were significantly decreased. Expression of MUC1 increases drug resistance in BC patients. In addition, MUC1 increases glycolysis, glucose uptake, and lactate production by inducing metabolic reprogramming.

**Conclusion:**

MUC1 has a significant effect on the metabolism and immune cell infiltration of BC, which may be the cause of increased drug resistance, and can be used as a molecular target for the diagnosis and treatment of BC.

## Introduction

Bladder cancer (BC) is a malignant tumor that seriously endangers human health. There are approximately 573,000 new cases of BC each year worldwide, and the number of deaths caused by BC is approximately 213,000 ranking tenth among all tumors [1]. BC is divided into muscle invasive bladder cancer (MIBC) and non-muscle invasive bladder cancer (NMIBC) according to the depth of invasion [2]. BC mainly includes transitional cell carcinoma, squamous cell carcinoma, and adenocarcinoma on the basis of the pathological type, among which transitional cell carcinoma accounts for more than 90% [3]. There are plenty of risk factors in the development of BC, including gender, smoking prevalence, occupational carcinogen exposure, *Schistosoma haematobium* infection, etc. [4]. Recently, researchers have considered the interaction of hypoxia and tumor, immune cell infiltration and immune microenvironment as the key step in the occurrence and development of BC [5, 6]. Currently, early diagnosis of BC can rely on serum markers, urine markers, and cystoscopy. However, the lack of clear markers and poor patients’ compliance make the early diagnosis of BC very difficult [7]. Despite the 5-year survival rate for BC is as high as 77%, the recurrence rate of BC is still high, and the 5-year survival rate for metastatic BC is less than 5% [8]. Therefore, it is particularly urgent to find highly sensitive biomarkers for BC.

MUC1 encodes a type I transmembrane protein with a polar distribution, called Mucin 1. N-terminal alpha subunit of Mucin1 functions in cell adhesion and the C-terminal beta subunit is involved in cell signaling [9]. Recent studies have found that MUC1 plays an important role in tumor hypoxia microenvironment and tumor metabolism [10]. MUC1 can not only mediate hypoxia-driven angiogenesis by regulating a variety of angiogenic factors; it can also stabilize and activate hypoxia inducible factor-1α (HIF-1α) to promote the metabolic reprogramming of cancer cells [11, 12]. Recently, studies have developed antibody–drug conjugated (ADC) based on MUC1 employed in the treatment trastuzumab-resistant breast cancer patients [13]. Besides, a multitargeted recombinant Ad5 PSA/MUC-1/brachyury-based immunotherapy vaccine developed by Bilusic et al. was recently introduced into phase I trials [14]. The types and abundance of immune cell infiltration in the tumor microenvironment have proved to be very different from normal tissues [14]. Studies have found that the expression of MUC1 induces immune suppression in colon cancer, and this suppression can be reversed by blocking the PD1/PDL1 pathway [15]. MUC1 has shown a strong potential in the diagnosis and treatment of tumors.

Although some studies claim that MUC1 has significance in BC, the specific mechanism remains unclear. In this study, we used gene expression data obtained from the the Cancer Genome Atlas (TCGA) and the Gene Expression Omnibus (GEO) database to explore the expression of MUC1 in human BC samples. We used R (version 4.0.3) to analyze the correlation between MUC1 expression and clinical characteristics. To better understand the mechanism of MUC1 in the occurrence and development of BC, we conducted Gene Set Enrichment Analysis (GSEA) and Gene Ontology (GO) and Kyoto Encyclopedia of Genes and Genomes (KEGG) analysis [16]. Next, we used Tumor Immune Estimation Resource (TIMER) and CIBERSORT to analyze the relationship between MUC1 expression and tumor immune cell infiltration. Cancer Cell Line Encyclopedia (CCLE) and Genomics of Drug Sensitivity in Cancer (GDSC) was used to analyze the relationship between MUC1 expression and drug sensitivity. Finally, we use Gene Expression Profile Interaction Analysis (GEPIA), Kaplan–Meier survival analysis (KM), and Human Protein Atlas (HPA) to analyze the relationship between MUC1 and patient prognosis.

## Materials and methods

### Data retrieval and download

To explore the expression of MUC1 in BC and its relationship with clinical characteristics, gene expression data and clinical data of BC in TCGA (TCGA-BLCA cohort) were downloaded from the Genomic Data Commons Data Portal (https://portal.gdc.cancer.gov). Besides, we downloaded the gene expression data and survival data of GSE31684 and GSE13507 as a validation dataset from the GEO (https://www.ncbi.nlm.nih.gov/geo) [17, 18].

### Gene expression analysis

We compared the expression of MUC1 in cancer and adjacent normal tissues, as well as in positive and negative lymphatic metastasis tissues. With the GSE13507 dataset, we compared MUC1 expression in normal, paraneoplastic, primary and recurrent tumour tissues. In addition, we used GEPIA to show the expression of MUC1 in bladder tumor tissues and adjacent tissues (http://gepia.cancer-pku.cn/) [19]. Next, we removed samples with incomplete clinical data (age, gender, American Joint Committee on Cancer (AJCC) pathologic stage, AJCC pathologic N, AJCC pathologic M, AJCC pathologic T). The protein expression of MUC1 between normal and cancer tissues was retrieved and compared from the HPA database (www.proteinatlas.org).

### Prognostic analysis

Univariate and multivariate analyses were conducted using the Cox Proportional Hazards Regression model on the remaining samples to obtain risk scores. To further understand the relationship between MUC1 expression and clinical characteristics, we conducted receiver operating characteristic (ROC) analysis and logistic regression analysis. After dividing the samples into high and low expression group according to the median expression of MUC1, KM survival analysis was conducted to analyze the overall survival (OS) rate of BC patients related to MUC1 expression. Furthermore, we conducted a similar analysis in GSE31684 and GES13507 to verify the results obtained previously.

### Gene set enrichment analysis

To explore the mechanism of MUC1 in BC, we divided the samples into MUC1-H and MUC1-L groups according to the level of MUC1 expression. Next, the gene differential expression analysis was performed on two sets of samples, and the genes that were differentially expressed were obtained (logFC > 2, *p* value < 0.05). Incorporate differentially expressed genes in GSEA analysis. The number of permutations was set to 1000. To investigate the possible biological functions of MUC1, we used GSEA to analyze the GO pathway and the KEGG pathway. The absolute value of normalized enrichment score (|NES|) is greater than 2, nominal *p* value (NOM *p* value) is less than 0.05, and false discovery rate (FDR) is less than 0.05 of all enrichment pathways.

### Immune infiltrate analysis

Li et al. developed a deconvolution algorithm (TIMER) to calculate the type and abundance of immune cell infiltration based on the gene expression profiles (http://timer.cistrome.org/) [20]. We evaluated MUC1 expression in BC and its correlation with the type and abundance of immune cell infiltration, including B cells, CD4^+^ T cells, CD8^+^ T cells, Dendritic cells, Neutrophils and Macrophages. Another deconvolution algorithm based on gene expression called CIBERSORT (http://cibersort.stanford.edu/) was used to further assess the relationship between MUC1 and immune cell [21, 22]. And we performed a nonparametric Wilcox test to assess whether the infiltration of immune cells differed between MUC1-H and MUC-L groups. In addition, we also assessed the correlation between different immune cells.

### Metabolism analysis

The samples in the TCGA, BLCA, and GSE13507 datasets were divided into two groups according to the expression of MUC1, and the expression levels of genes related to glucose metabolism (HK2, GLUT1, PKM, RWDD3, SLC16A3, SLC5A12, ENO1) in different groups were compared. Data were analyzed using t-test and *p* value < 0.05 was considered statistically significant.

### Drug sensitivity analysis

CCLE/GDSC (https://public.tableau.com/app/profile/jason.roszik/viz/CCLE_GDSC_correlations/CCLE_GDSC) was used to analyze the relationship between MUC1 expression and drug sensitivity. Retrieve and select MUC1 in the gene list, and the coefficient concentration was adjusted as IC50 values. Spearman’s coefficient (ρ) ≥ 0.5 and *p* values < 0.05 were considered associated with MUC1.

### Statistical analysis

All statistical analyses were conducted using SPSS version 22.0 or R version 4.0.3 with following packages: ‘caret’, ‘survival’, ‘magrittr’, ‘ggplot2’, ‘ggpubr’, ‘survminer’, ‘survivalROC’, ‘limma’, ‘org.Hs.eg.db’, ‘clusterProfiler’, ‘enrichplot’ and ‘corrplot’. Univariate and multivariate analyses with Cox proportional hazards regression for OS were performed and hazard ratios (HR) and 95% confidence intervals (CI) were estimated. Logistic regression was used to test the relationship between MUC1 expression and clinical features with age, gender, AJCC pathologic stage, AJCC pathologic N, AJCC pathologic M, AJCC pathologic T as covariates. All hypotheses were two-sided and *p* value < 0.05 was considered statistically significant.

## Results

### Expression of MUC1 at the mRNA and protein levels and its relationship to clinical characteristics

**Fig. 1:**
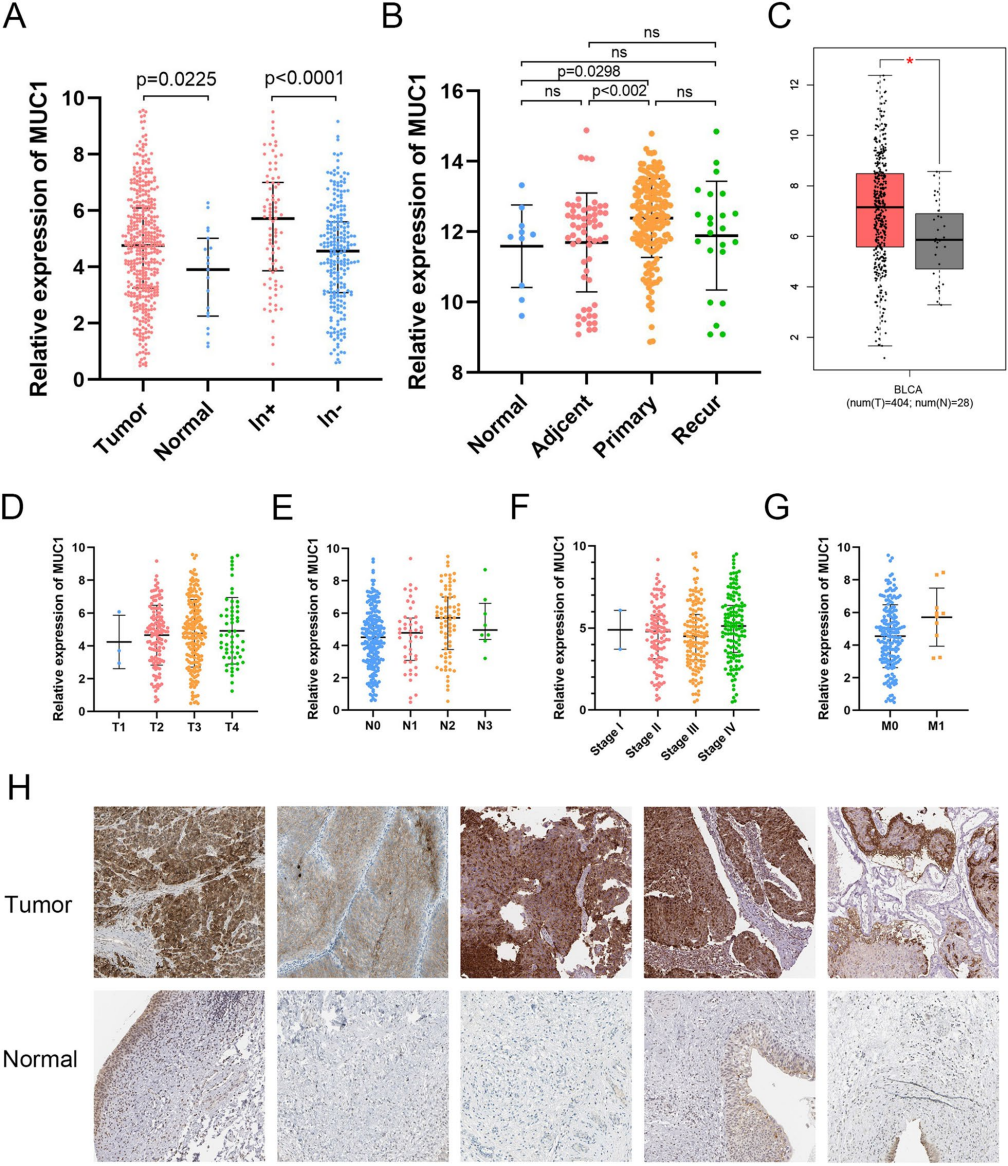
Gene expression analysis. **A** Expression of MUC1 in bladder tissue of TCGA (tumor vs. normal, lymph node metastasis positive vs. lymph node metastasis negative). **B** Expression of MUC1 in bladder tissue of GSE133507 (normal vs. adjcent vs. primary vs. recer). **C** Comparison of MUC1 expression in tumor and normal tissues through the GEPIA database. **D**–**G** Relationship between MUC1 expression and clinical characteristic(Stage, T stage, M stage and N stage). **H** Expression of MUC1 in BC and adjacent tissues by immunohistochemistry (HPA)

We compared MUC1 mRNA expression between BC and normal tissues in TCGA database, and also between lymphphatic metastasis negative and positive samples. A total of 411 tumor tissues and 19 normal tissues were enrolled in the expression analysis, including 233 lymphatic metastasis negative and 81 lymphatic metastasis positive samples. The expression of MUC1 in different groups was represented by Scatterplot (Fig. 1A). The results showed that the expression of MUC1 in cancer tissues was significantly higher than that in normal tissues (*p* value = 2.2e−2), and it was significantly higher in lymph node metastasis positive samples than in lymph node metastasis negative (*p* value = 4.2e−5). The GSE13507 dataset contains normal, paraneoplastic, primary and recurrent tumour samples. We analysed the expression of MUC1 in different subgroups of GSE13507. The results showed significant differences in MUC1 between primary tumours and paraneoplastic tissues (*p* value < 0.002). Similarly, MUC1 was significantly different between primary tumours and normal tissues (*p* value = 0.0298) (Fig. 1B). Furthermore, we used GEPIA to compare the expression of MUC1 in normal and tumor tissues and found that the mRNA expression of MUC1 significantly increased in tumor tissues (*p* value < 0.01) (Fig. 1C).

We evaluated the connection between clinical characteristics and MUC1 expression levels of BC patients. There are 382, 382, 192, 350 samples with complete data of AJCC pathologic Stage, AJCC pathologic T stage, AJCC pathologic M stage, and AJCC pathologic N stage, respectively. The results showed that MUC1 has little correlation with AJCC pathological T stage (*p* value > 0.05, Fig. 1D) and AJCC pathological Stage (*p* value > 0.05, Fig. 1F), but is significantly correlated with AJCC pathological N stage (*p* value < 0.05, Fig. 1E) and AJCC pathological M stage (*p* value < 0.05, Fig. 1G). In addition, we conducted logistic regression to analyze the correlation between MUC1 expression and clinical characteristics (Table 1). The increase of MUC1 expression level in BC was significantly correlated with lymphatic metastasis (N1 vs. N0, *p* value = 0.007). These indicate that compared with BC patients with low levels of MUC1 expression, BC patients with high levels of MUC1 expression are more likely to develop lymphatic metastasis.

**Table 1:** Association between MUC1 expression and clinicopathologic characteristics using logistic regression

Clinical characteristic	B	S.E.	Wald	Odds ratio in MUC1 expression	*p* value
Age	− 0.02	0.013	2.544	0.98 (0.956–1.005)	0.111
Gender (female vs. male)	− 0.156	0.338	0.211	0.856 (0.441–1.662)	0.646
Stage (II vs. I)	− 1.872	1.686	1.233	0.154 (0.006–4.19)	0.267
Stage (III vs. I)	− 2.352	1.791	1.726	0.095 (0.003–3.181)	0.189
N (N1 vs. N0)	1.457	0.545	7.154	4.292 (1.476–12.48)	0.007
N (N2 vs. N0)	− 1.324	1.723	0.591	0.266 (0.009–7.787)	0.442
N (N3 vs. N0)	− 2.661	1.723	2.386	0.07 (0.002–2.045)	0.122
N (N4 vs. N0)	− 2.162	2.006	1.162	0.115 (0.002–5.867)	0.281
M (M1 vs. M0)	0.179	0.292	0.374	1.196 (0.674–2.12)	0.541
M (M2 vs. M0)	− 0.662	1.149	0.332	0.516 (0.054–4.901)	0.564
T (T4 vs. T1)	− 0.811	0.79	1.053	0.444 (0.094–2.092)	0.305
T (T4 vs. T1)	− 0.507	0.472	1.153	0.602 (0.239–1.52)	0.283

Next, to understand the difference in protein levels of MUC1, we searched HPA for the immunohistochemical data of MUC1 in BC. The results showed that MUC1 has higher levels of expression in tumor compared to normal tissues (Fig. 1H).

### The role of MUC1 in the prognosis of bladder cancer

**Fig. 2:**
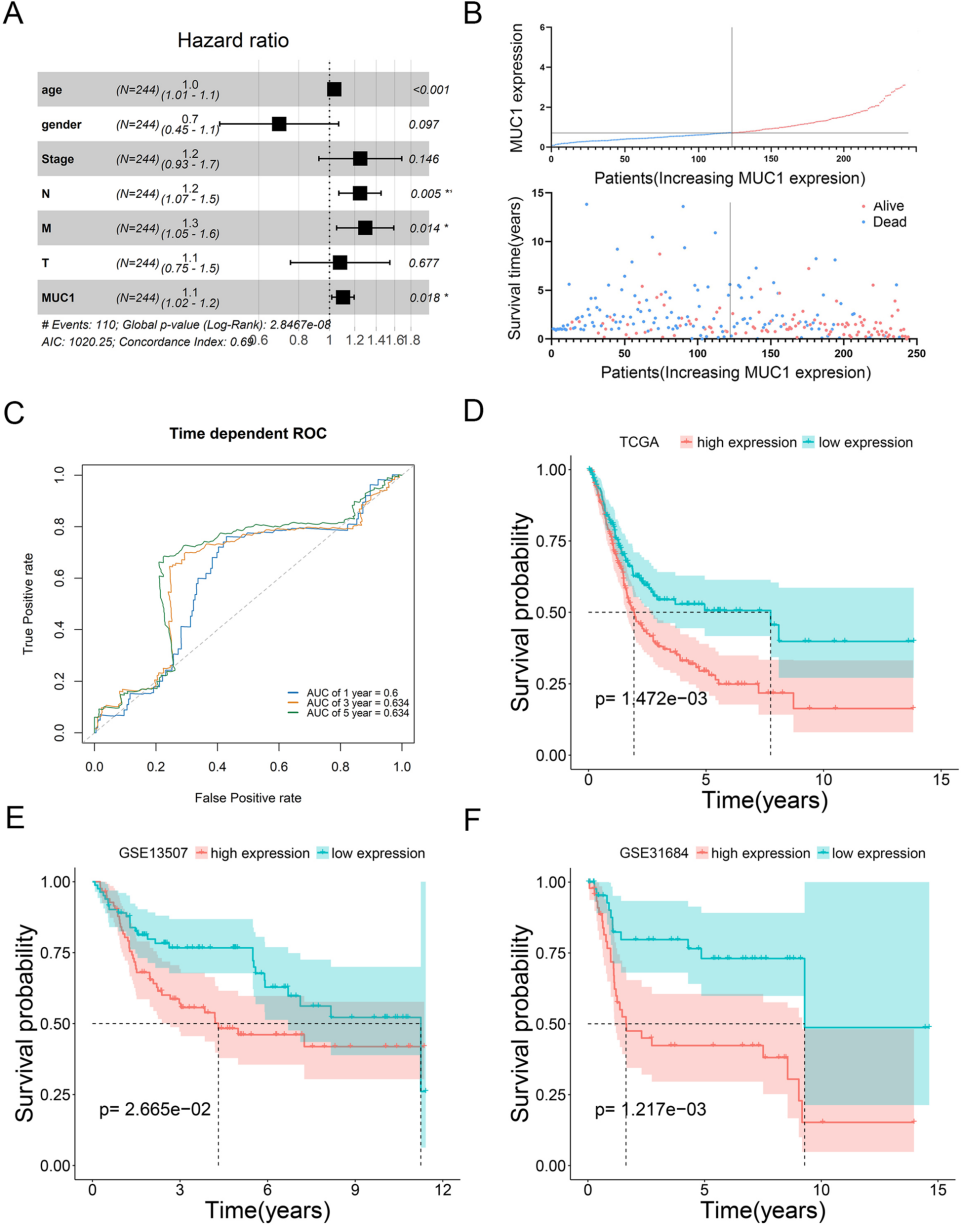
Prognostic analysis. **A** Multivariate Cox clinical independence prognostic analysis. **B** Distribution of MUC1 expression in TCGA and survival status of BC patients. **C** Multiple ROC curves based on MUC1 expression and clinical characteristic (1-year, 3-year, 5-year). **D**–**F** Survival analysis according to the level of MUC1 expression (**D** TCGA_BLCA, **E** GSE31684, **F** GSE13507)

We used univariate Cox proportional hazards regression analysis to explore the relationship between MUC1 expression, clinical characteristics, and OS. The results showed that Age (HR = 1.03 (1.01–1.05), *p* value = 2.41e−03), AJCC pathologic Stage (HR = 1.56 (1.23–1.99), *p* value = 3.08e−04), AJCC pathologic N (HR = 1.24 (1.09–1.4), *p* value = 9.3e−04), AJCC pathologic M (HR = 1.34 (1.11–1.63), *p* value = 2.42e−03), AJCC pathologic T (HR = 1.33 (1.01–1.76), *p* value = 4.34e−02), MUC1 (HR = 1.16 (1.07–1.26), *p* value = 5.23e−04) were significantly related to OS (Table 2). Next, multivariate Cox proportional hazards regression analysis showed that MUC1 expression is an independent prognostic factor (Fig. 2A, Table 3). The distribution of MUC1 expression, survival status of patients with BC, and expression profiles of MUC1 are shown in Fig. 2B. ROC curve analysis showed that the AUC values of 1, 3, and 5 years were 0.6, 0.634, and 0.634, respectively, indicating that the expression of MUC1 has a potential prognostic ability (Fig. 2C).

**Table 2:** Correlation between overall survival and multivariable characteristics in TCGA patients via Univariate Cox regression model

ID	Coef	HR	HR.95L	HR.95H	*p* value
Age	0.0294	1.0299	1.0105	1.0496	0.0024
Gender	− 0.4055	0.6667	0.4378	1.0153	0.0588
AJCC_pathologic_Stage	0.4458	1.5617	1.2258	1.9895	0.0003
AJCC_pathologic_N	0.2126	1.2369	1.0906	1.4028	0.0009
AJCC_pathologic_M	0.2964	1.3450	1.1106	1.6288	0.0024
AJCC_pathologic_T	0.2864	1.3317	1.0085	1.7584	0.0434
MUC1	0.1465	1.1578	1.0658	1.2578	0.0005

**Table 3:** Correlation between overall survival and multivariable characteristics in TCGA patients via multivariate Cox regression model

ID	Coef	HR	HR.95L	HR.95H	*p* value
Age	0.0340	1.0345	1.0142	1.0553	0.0008
Gender	− 0.3637	0.6951	0.4523	1.0682	0.0971
AJCC_pathologic_Stage	0.2218	1.2483	0.9256	1.6836	0.1461
AJCC_pathologic_T	0.2200	1.2461	1.0699	1.4513	0.0047
AJCC_pathologic_T	0.2576	1.2938	1.0526	1.5902	0.0144
AJCC_pathologic_T	0.0763	1.0793	0.7540	1.5451	0.6766
MUC1	0.0973	1.1022	1.0165	1.1950	0.0184

Next, we performed a survival analysis in TCGA based on the expression of MUC1. The results of TCGA show that MUC1 expression is significantly related to OS (*p* value = 1.47e−03) (Fig. 2D). To further verify the influence of MUC1 on the OS of BC patients, we further conducted similar analysis in GSE13507 and GSE31684. As expected, similar results appeared in GSE31684 (*p* value = 1.21e−03) and GSE13507 (*p* value = 2.66e−02) (Fig. 2E, F).

### MUC1 related enrichment pathway

**Fig. 3:**
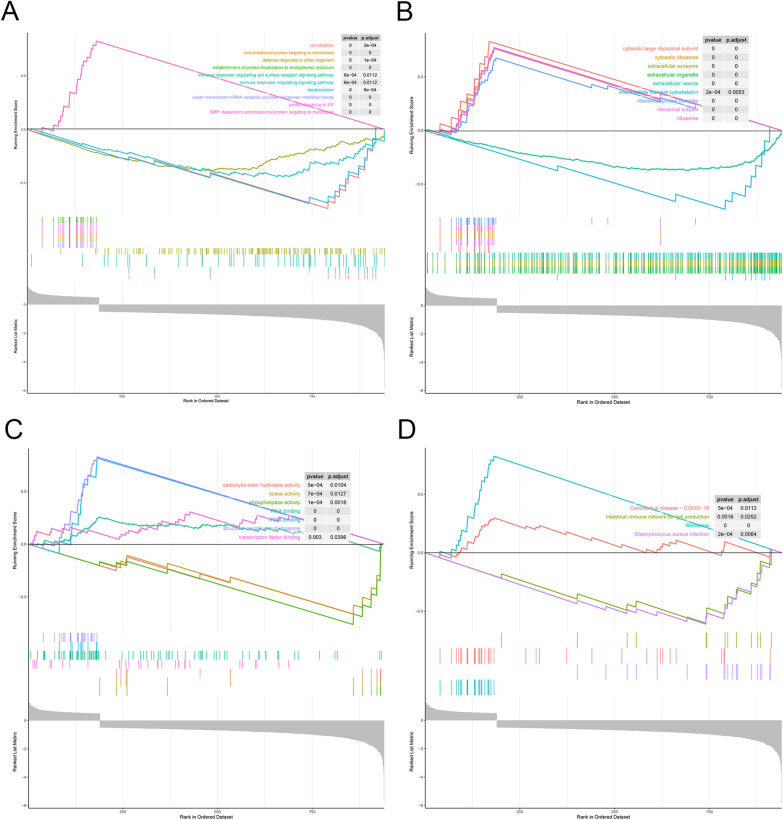
Gene set enrichment analysis. **A** GO biological process terms. **B** GO Cellular component terms. **C** GO molecular function terms. **D** KEGG terms

To explore the potential biological functions of MUC1, we performed GO term and KEGG pathway enrichment analyses of differentially expressed genes. Signal pathways that meet the following conditions (|NES| > 2, NOM *p* value < 0.05, FDR < 0.05) are considered enriched. As shown in Table 4, there are five positive GO Biological Process terms related to MUC1 (nuclear-transcribed mRNA catabolic process, nonsense-mediated decay; cotranslational protein targeting to the membrane; SRP-dependent cotranslational protein targeting to the membrane; protein targeting to ER; establishment of protein localization to endoplasmic reticulum) and five negative GO Biological Process terms related to MUC1 (cornification; keratinization; immune response-regulating signaling pathway; immune response-regulating cell surface receptor signaling pathway; defense response to other organism) (Fig. 3A). There are five positive GO Cellular Component terms related to MUC1 (cytosolic large ribosomal subunit; cytosolic ribosome; ribosomal subunit; ribosome; ribonucleoprotein complex) and four negative GO Cellular Component terms related to MUC1 (intermediate filament cytoskeleton; extracellular organelle; extracellular exosome; extracellular vesicle) (Fig. 3B). There are three positive GO Molecular Function terms related to MUC1 (RNA binding; rRNA binding; structural constituent of ribosome) and four negative GO Molecular Function terms related to MUC1 (phospholipase activity; lipase activity; carboxylic ester hydrolase activity; antigen binding) (Fig. 3C). As shown in Table 5, there are two positive KEGG pathways related to MUC1 (Coronavirus disease, COVID-19 and Ribosome) and two negative KEGG pathways related to MUC1 (*Staphylococcus aureus* infection and Intestinal immune network for IgA production) (Fig. 3D). These results indicate that the pathways that regulate ribosomal and lipid metabolism are critical to BC patients and are closely related to the expression of MUC1.

**Table 4:** GO signaling pathways most significantly correlated with MUC1 expression

	GO ID	GO name	NES	NOM *p* value	p.adjust	FDR q-value
Negative	GO:0070268	Cornification	− 2.4033	8.33E−06	0.0003	0.0002
	GO:0004620	Phospholipase activity	− 2.3010	4.52E−05	0.0011	0.0009
	GO:0031424	Keratinization	− 2.2808	1.97E−05	0.0005	0.0005
	GO:0045111	Intermediate filament cytoskeleton	− 2.2227	0.0001	0.0029	0.0024
	GO:0016298	Lipase activity	− 2.1578	0.0003	0.0060	0.0051
Positive	GO:0072599	Establishment of protein localization to endoplasmic reticulum	4.40526	1.00E−10	1.30E−08	1.11E−08
	GO:0022626	Cytosolic ribosome	4.4063	1.00E−10	1.30E−08	1.11E−08
	GO:0005840	Ribosome	4.7311	1.00E−10	1.30E−08	1.11E−08
	GO:0003735	Structural constituent of ribosome	4.7678	1.00E−10	1.30E−08	1.11E−08
	GO:1990904	Ribonucleoprotein complex	4.8974	1.00E−10	1.30E−08	1.11E−08

**Table 5:** KEGG signaling pathways most significantly correlated with MUC1 expression

	KEGG ID	KEGG name	NES	NOM *p* value	p.adjust	FDR q-value
Negative	hsa05150	*Staphylococcus aureus* infection	− 2.2056	0.0002	0.0064	0.0043
	hsa04672	Intestinal immune network for IgA production	− 2.0161	0.0016	0.0252	0.0171
Positive	hsa05171	Coronavirus disease—COVID-19	2.1688	0.0005	0.0112	0.0076
	hsa03010	Ribosome	4.7547	1.00E−10	6.20E−09	4.21E−09

### Relationship between MUC1 expression and immune cell infiltration

**Fig. 4:**
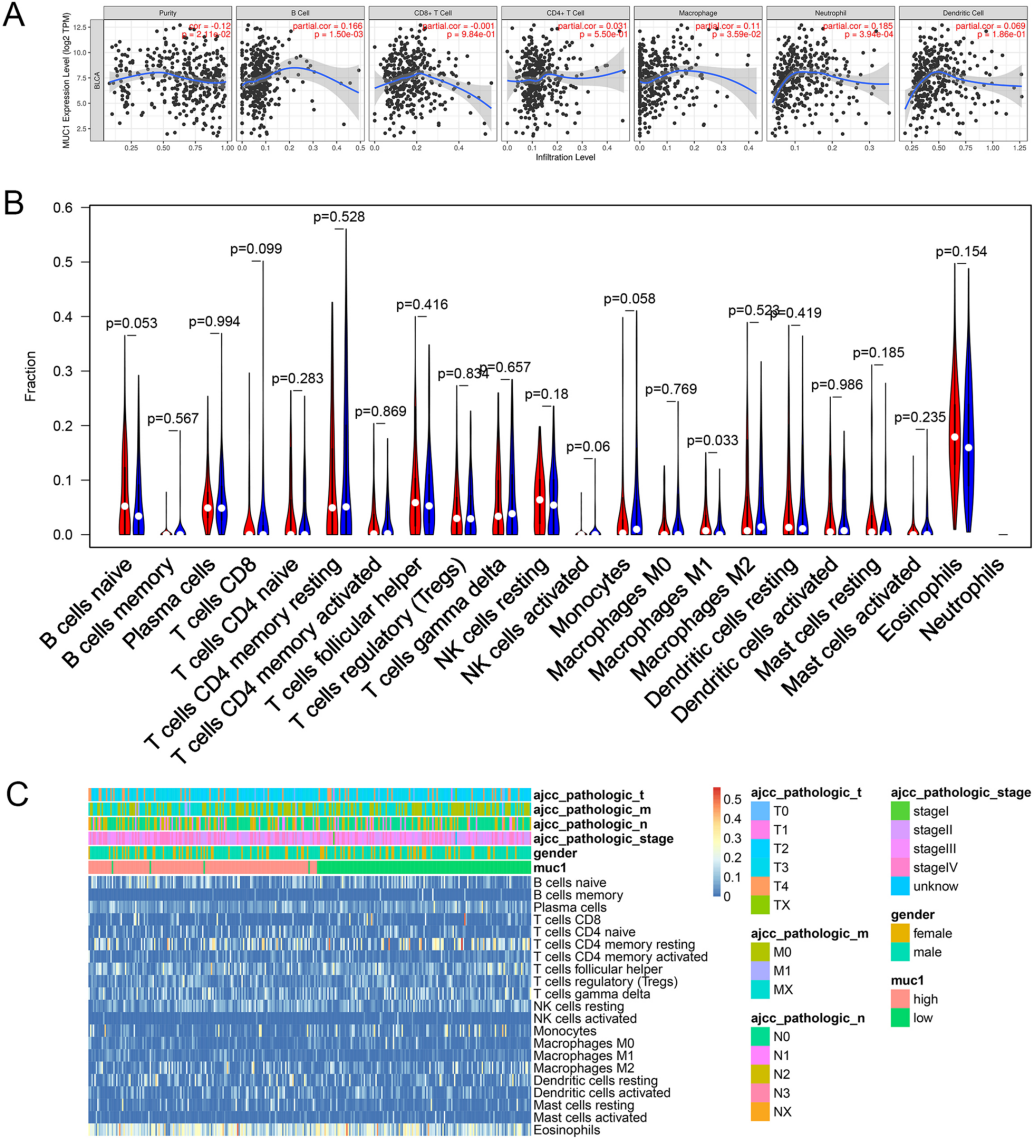
Immune infiltrates analysis. **A** The correlation between the expression of MUC1 and the level of BC immune cell infiltration (TIMER). **B** The relationship between immune cell infiltration abundance and MUC1 expression (CIBERSORT). **C** The correlation of immune cell infiltration to clinical characteristics

We used TIMER to clarify the correlation between the expression of MUC1 and the level of immune infiltration of BC. The results have shown that MUC1 expression is significantly correlated with B cells (*p* value = 1.50e−03), macrophages (*p* value = 3.593e−02) and Neutrophils (*p* value = 3.94e−04), indicating that MUC1 plays a key role in the immune infiltration of BC (Fig. 4A). To explore the impact of MUC1 expression on the immune microenvironment of BC patients, we divided the samples into MUC1-H and MUC1-L according to MUC1 expression. CIBERSORT was used to calculate the infiltration abundance of 22 immune cells in each sample to assess the difference between different groups. The results indicate that M1 macrophages are affected by the expression of MUC1 (*p* value = 0.033) (Fig. 4B). We also assessed the potential correlation between immune cell infiltration and clinical features. The results showed a strong correlation between immune cell infiltration and clinical features (Fig. 4C).

### Relationship between MUC1 expression and metabolism

**Fig. 5:**
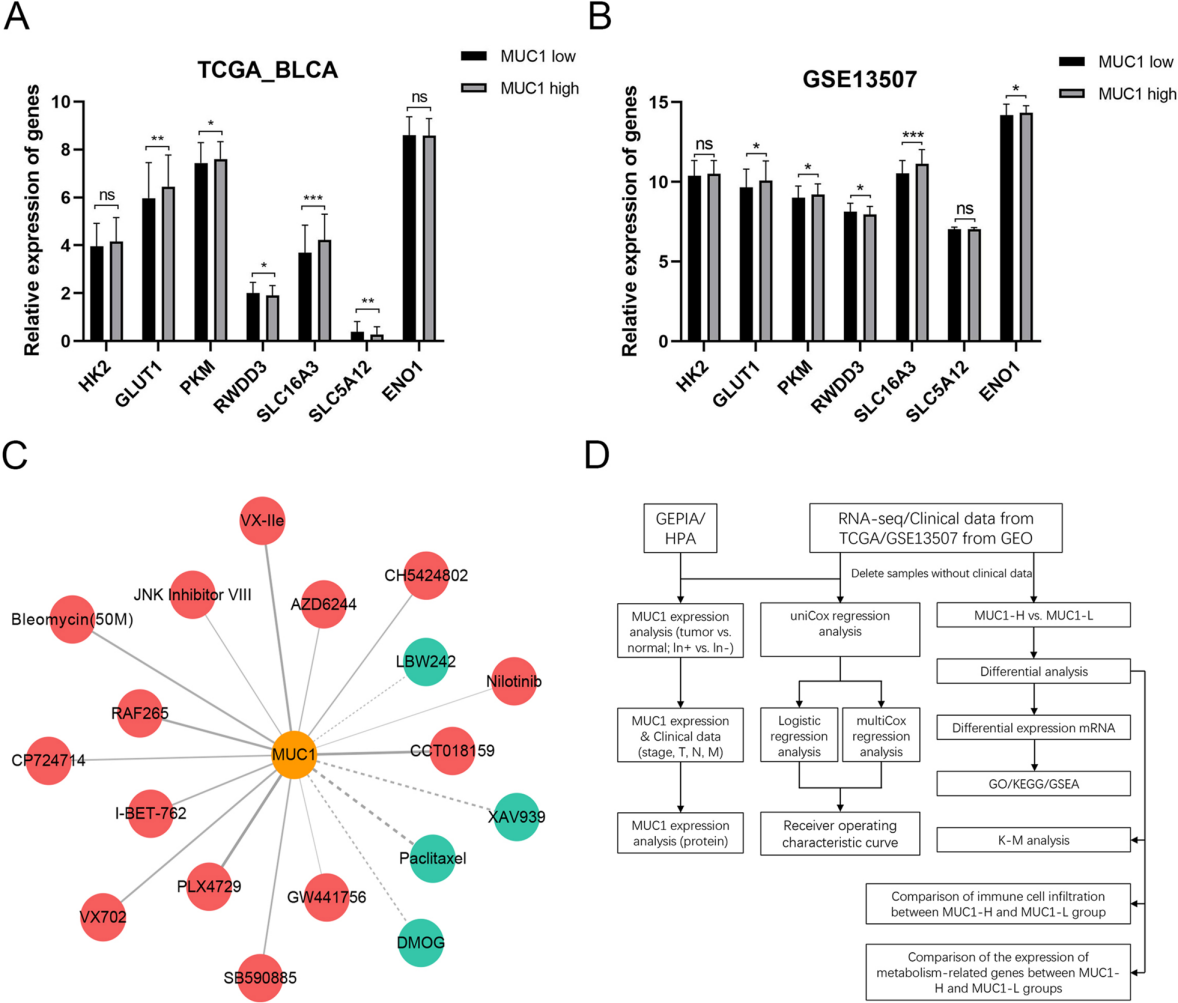
Metabolism and drug sensitivity analysis. **A** Metabolism-related genes expression differences whthin TCGA_BLCA between the MUC1-L and MUC1-H groups. **B** Expression differences of metabolism-related genes in GSE13507 between the MUC1-L and MUC1-H groups. **C** Relationship between MUC1 expression and drug sensitivity. (Red and solid lines represent drug-resistant drugs, and cyan and dotted lines represent effectiveness-enhancing drugs. The width and transparency of the line represent the force of the correlation.) **D** Schematic diagram of the analysis process

To explore the relationship between MUC1 and metabolism, we analyzed the relationship between MUC1 expression and major metabolism-related genes in the TCGA-BLCA cohort and GSE13507 dataset. The results showed that MUC1 significantly increased the expression of GLUT1 (*p* value = 6.45e−04), PKM (*p* value = 3.75e−02) and SLC16A3 (*p* value = 1.73e−02), while the expression of RWDD3 (*p* value = 3e−06) was significantly decreased (Fig. 5A). The same result appeared in GSE13507 (GLUT1 (*p* value = 5.21e−03), PKM (*p* value = 2.46e−02), SLC16A3 (*p* value = 4.90e−03) and RWDD3 (*p* value = 1e−06)). This implies that MUC1 may enhance glucose metabolism. Interestingly, in the GSE13507 dataset, we also observed that ENO1 (*p* value = 3.69e−02) was significantly increased in the MUC1 high expression group, which suggests that the increase of MUC1 may lead to the enhancement of aerobic glycolysis (Fig. 5B).

### Relationship between MUC1 expression and drug sensitivity of BC patients

In CCLE database, MUC1 expression significantly decreased the IC50 efficacy of MEK inhibitors AZD6244, Bcr/Abl inhibitors Nilotinib, Raf inhibitors PLX4729, and Raf kinase inhibitors RAF265; but increased the efficacy of IAP inhibitors LBW242 and Paclitaxel (Table 6). The positive values of the Spearman coefficient (ρ) indicate resistance while a negative correlation indicates an enhancing effect on drug efficacy. The expression of MUC1 increases the resistance of most drugs in GDSC, including Bleomycin (50 μM), CCT018159, CH5424802, CP724714, GW441756, I-BET-762, JNK Inhibitor VIII, SB590885, VX-IIe, VX702. The targets of these drugs include DNA damage, HSP90, ERBB2, JNK, BRAF, ERK and p38, etc. (Table 6). Overall, MUC1 expression enhanced drug resistance in BC (Fig. 5C).

**Table 6:** Analysis of MUC1 expression and CCLE/GDSC drug sensitivity correlation in BLCA

	Drug	Target (s)	Spearman coefficient (ρ)	*p* value
CCLE	PLX4729	RAF	1	< 0.001
	RAF265	Raf kinase B, KDR	0.68	0.011
	AZD6244	MEK	0.63	0.035
	Nilotinib	Abl/Bcr-Abl	0.56	0.046
	LBW242	IAP	− 0.9	0.037
	Paclitaxel	Beta-tubulin	− 0.723	0.004
GDSC	Bleomycin (50 μM)	DNA damage	0.558	0.016
	CCT018159	HSP90	0.63	0.004
	CH5424802	ALK	0.5	0.029
	CP724714	ERBB2	0.504	0.027
	GW441756	NTRK1	0.543	0.045
	I-BET-762	BRD2, BRD3, BRD4	0.518	0.023
	JNK Inhibitor VIII	JNK	0.56	0.037
	SB590885	BRAF	0.579	0.023
	VX-IIe	ERK	0.581	0.009
	VX702	p38	0.618	0.019
	DMOG	Prolyl-4-Hydroxylase	− 0.505	0.027
	XAV939	TNKS1, TNKS2	− 0.556	0.017

## Discussion

Recently, the role of MUC1 in tumors has gradually been emphasized. In pancreatic cancer, MUC1 reduces radiation-induced pancreatic cancer cell toxicity and DNA damage by enhancing glycolysis, the pentose phosphate pathway, and nucleotide biosynthesis [23]. In triple-negative breast cancer, MUC1-C increases PD-L1 transcription and promotes tumor immune escape by recruiting MYC and NF-κB p65 to the PD-L1 promoter region [24]. In prostate cancer, MUC1-C inhibits androgen receptor (AR) axis signaling and induces the expression of the neural BRN2 transcription factor, increasing the self-renewal capacity and tumorigenicity of prostate cancer cells [25]. In addition, MUC1 also plays an important role in ovarian cancer [26], non-small cell lung cancer [27] and pancreatic cancer [28]. One study found that the positive rates of MUC1 and MUC2 in urothelial carcinoma were 89.7% and 44.3%, respectively, and MUC1 expression was significantly correlated with tumor grade, while MUC2 was the opposite. Different staining patterns of MUC1 and MUC2 have important prognostic implications in patients with difficult-to-recognize low-grade papillary urothelial lesions and precancerous lesions [29]. Urothelial carcinoma patients expressing high levels of MUC1-C have poor survival and are strongly associated with cisplatin resistance [30]. However, the specific mechanism of MUC1 in BC remains unclear. We analyzed the potential mechanism of MUC1 in BC (Fig. 5D).

In our current study, to explore the possibility of MUC1 as a prognostic biomarker for BC, we used TCGA BLCA cohort to evaluate the prognostic value of MUC1. First, we analyzed the expression of MUC1 (protein level and mRNA level), and the results showed that the expression of MUC1 in tumor tissues was significantly higher than that in adjacent normal tissues, and the expression of MUC1 in lymphatic metastasis positive samples was significantly higher than that in lymphatic metastasis negative samples. BC patients with high expression of MUC1 showed advanced N stage, M stage, and tumor status. We analyzed the role of MUC1 and clinical features in prognosis, and the results showed that high MUC1 expression was strongly associated with worse prognosis. These results suggest that MUC1 may serve as an independent prognostic factor for overall survival in BC.

MUC1 is closely related to metabolism and resulting drug resistance. Recent studies have found that there is a crosstalk between the MUC1 and HIF-1 signal pathways. The HIF-1 signaling pathway has been shown to be abnormally activated in numerous cancers, especially solid tumors [31]. HIF-1 consists of two subunits, HIF-1α and HIF-1β, of which the α subunit is the active unit. In oxygenated cells, HIF-1α will be hydroxylated by proline hydroxylase (PHD1), which will then be recognized and degraded by the ubiquitin-protease complex; while in hypoxic cells, the activity of PHD1 decreases and the lack of a substrate for the hydroxylation reaction leads to the accumulation of HIF-1α [32]. The transcriptional targets of HIF-1α include genes that play key roles in angiogenesis, erythropoiesis, metabolic reprogramming, vasomotor function, and apoptosis proliferation response [33]. In the hypoxic condition, MUC1 promotes the recruitment of HIF-1α and p300 to the promoter region of glycolysis genes, upregulates the expression of glycolysis-related genes, increases glucose uptake and lactate production, and assists tumor cells to survive in hypoxic environments [12]. In addition, MUC1 can increase the carbon flux of the non-oxidative pentose phosphate pathway (PPP) and pyrimidine synthesis pathway of pancreatic cancer cells by promoting the expression of HIF-1α, leading to gemcitabine resistance [34].

In this study, GLUT1, PKM, and SLC16A3 were significantly upregulated in the MUC1-H group, while RWDD3 was significantly downregulated. GLUT1 encodes the major glucose transporter in cell [35]. Pyruvate kinase is encoded by the PKM and is the main rate-limiting enzyme in the glycolysis process [36]. SLC16A3 removes lactate produced by glycolysis from the plasma membrane and plays a key role in the growth and proliferation of hypoxic cancer cells [37]. RWDD3 negatively regulates the HIF-1α signaling pathway by increasing the sumoylation of HIF-1α, promoting its stabilization, transcriptional activity, and the expression of its target gene VEGFA during hypoxia [38]. Therefore, we can draw the hypothesis: MUC1 increases the uptake of glucose and the production of lactate through the HIF-1α signaling pathway, so that tumor cells can survive under hypoxic conditions. In addition, GO and KEGG pathway enrichment analysis also showed that up-regulated MUC1 was mainly closely related to metabolism. Through the CCLE and GDSC databases, we found that the expression of MUC1 in BC was associated with drug resistance, which may be caused by increased pyrimidine metabolism, and the specific mechanism remains to be further studied. We used the TIMER database to explore the relationship between the expression of MUC1 and the level of immune infiltration in BC. The results showed that MUC1 was significantly related to B cell, Macrophages, and Neutrophils. In addition, we further verified this result with CIBERSORT. The results showed that the level of M1 macrophages was significantly reduced in the MUC1-H group. Our results indicate that MUC1 may be involved in the regulation of M1 macrophages in BC. Macrophages are a very heterogeneous cell population with a series of continuous functional states in the body, and M1 and M2 macrophages are the two extremes of this continuous state. M1 macrophages participate in the positive immune response and perform the function of immune surveillance by secreting proinflammatory cytokines and chemokines and present antigens on a full-time basis; M2 macrophages have weak antigen presentation ability. It plays an important role in immune regulation by secreting the inhibitory cytokine IL-10 or TGF-B, etc. [39].

There is a very important immunotherapy for the treatment of BC: BCG. High expression of MUC1 enhances BCG-induced immune response. He et al. constructed a recombinant Bacillus Calmette Guérin (rBCG)-MUC1-interleukin-2 (IL-2), and in vivo experiments showed that rBCG-MUC1-IL2 can preferentially induce MUC1-specific cellular immune responses and can be used as a vaccine for the prevention and treatment of breast cancer [40]. Almost simultaneously, Maureen et al. successfully constructed a BCG-human interleukin-2 (hIL2)-MUC1 breast cancer vaccine, which significantly reduced the incidence and rate of tumorigenesis in mice, suggesting that co-expression of MUC1 and IL-2 plays a key role in the induction of tumor-specific immune responses [41]. Yuan et al. constructed a BCG-MUC1-based breast cancer vaccine (rBCG-MVNTR4-CD80), which significantly suppressed tumors, induced interferon production, and stimulated both CD4+ and CD8+ positive lymphocyte production [42]. In addition, the authors constructed two other rBCG-MUC1 vaccines (rBCG-MVNTR4-CSF and rBCG-MVNTR8-CSF, with similar effects [43]. In 2016, an anti-tumor vaccine based on MUC1 (MUC1-maltose-binding protein (MBP)/BCG) entered preclinical trials and showed no significant toxicity in mice, rats or crab-eating monkeys.MUC1-MBP/BCG acts as an anti-tumor agent by inducing Th1 cell activation and MUC1-specific IgG antibody secretion [44]. At present, most of the research on MUC1 and BCG is targeting MUC1 and building tumor vaccines based on BCG to better play the role of BCG. Therefore, high MUC1 expression can theoretically improve the responsiveness of tumors to such rBCG drugs, but there is no research on the relationship between traditional BCG and MUC1 expression. This needs further study.

MUC1 has shown great potential as a widely expressed membrane protein in the diagnosis and treatment of tumors. However, the mechanism of action of MUC1 in BC has not been reported in the literature. The role of MUC1 in BC was investigated by expression analysis, prognostic analysis, immunological analysis, metabolic analysis and drug sensitivity analysis. The potential mechanisms of MUC1-mediated drug resistance in bladder cancer were uncovered. However, there are some shortcomings in this study: no experimental study was conducted to verify the function of MUC1, and the role of MUC1 needs to be verified by cellular and animal experiments subsequently.

## Conclusion

In short, this is the first report to explore the possibility of MUC1 as a marker of BC at the mechanistic level. This work helps to elucidate the role of cellular metabolism, immune cell infiltration, and genes regulating these processes in BC development and drug resistance. In addition, MUC1 can be used as a target for monoclonal antibodies, antibody–drug conjugate, cancer vaccine, Aptamer, and radiotherapy. Understanding the mechanism of MUC1 will help to develop corresponding treatment and diagnosis methods.

## Data Availability

The datasets analysed during the current study are available in the TCGA and GEO repository. The link is as follows: TCGA_BLCA: https://portal.gdc.cancer.gov; GSE13507: https://www.ncbi.nlm.nih.gov/geo/query/acc.cgi?acc=GSE13507; GSE31684: https://www.ncbi.nlm.nih.gov/geo/query/acc.cgi?acc=GSE31684.
